# Angiotensin-Converting Enzyme Related-Polymorphisms on Inflammation, Muscle and Myocardial Damage After a Marathon Race

**DOI:** 10.3389/fgene.2019.00984

**Published:** 2019-10-25

**Authors:** Ana Paula Rennó Sierra, Giscard Humberto Oliveira Lima, Elton Dias da Silva, Jaqueline Fernanda de Souza Maciel, Marino Pereira Benetti, Rodrigo Assunção de Oliveira, Patrícia Fátima de Oliveira Martins, Maria Augusta Pedanti Kiss, Nabil Ghorayeb, Philip Newsholme, João Bosco Pesquero, Maria Fernanda Cury-Boaventura

**Affiliations:** ^1^School of Physical Education and Sport, University of São Paulo, Sao Paulo, Brazil; ^2^Sports Cardiology Department, Dante Pazzanese Institute of Cardiology, Sao Paulo, Brazil; ^3^Department of Biophysics, Federal University of Sao Paulo, Sao Paulo, Brazil; ^4^Institute of Physical Activity and Sports Sciences, Cruzeiro do Sul University, Sao Paulo, Brazil; ^5^School of Pharmacy and Biomedical Sciences, Curtin Health Innovation Research Institute, Curtin University, Perth, WA, Australia

**Keywords:** skeletal muscle injury, exercise, angiotensinogen, bradykinin B2 receptor, myocardial injury, cytokines, genetic variation

## Abstract

Muscle damage is one of the most important factors that affect muscle fatigue during endurance exercise. Recent evidence suggests that the renin–angiotensin system impacts on skeletal muscle wasting. The aim of this study was to determine association between the *AGT* Met235Thr, *ACE* I/D and *BDKRB2* −9/+9 polymorphisms with inflammation, myocardial and muscle injury induced by endurance exercise. Eighty-one Brazilian male runners participated in this study and completed the International Marathon of Sao Paulo. Muscle and myocardial damage markers (alanine transaminase, ALT, aspartate transaminase, AST, lactic dehydrogenase, LDH, creatine kinase, CK, Troponin, pro BNP, myoglobin, and CK-MB) and inflammatory mediators (IL-6, IL-8, IL-10, IL12p70, IL1β, and TNF-α) were determined one day before, immediately after, one day after, and three days after the event. Muscle damage was also determined fifteen days after race and angiotensinogen (*AGT*) Met235Thr, angiotensin-converting enzyme (*ACE*) I/D, and Bradykinin B2 receptor (*BDKRB2*) −9/+9 polymorphisms were determined. Marathon race participation induced an increase in all muscle damage and inflammatory markers evaluated (p < 0.0001). The muscle damage markers, troponin and pro BNP, CK and LDH and inflammatory markers, IL-6, IL-8, IL-1β and IL-10 were also higher in *ACE* II genotype immediately after race, compared to DD genotype. The percentage of runners higher responders (>500U/I) to CK levels was higher for II genotypes (69%) compared to DD and ID genotypes (38% and 40%, respectively) immediately after. Troponin, pro BNP and IL-1β, IL-8 levels were also elevated in *AGT* MM genotype compared to TT genotype athletes after and/or one day after race. *BDKRB2* −9/−9 had pronounced response to LDH, CK, CK-MB and ALT and AST activities, myoglobin, troponin, IL-6, IL-8 levels immediately, one day and/or three days after race. The percentage of runners higher responders (>500U/I) to CK levels was greater for −9−9 and −9+9 genotypes (46 and 48%, respectively) compared to +9+9 genotypes (31%) immediately after. *ACE* II, *AGT* MM, and *BDKRB2* −9−9 genotypes may increase the susceptibility to inflammation, muscle injury after endurance exercise and could be used to predict the development of clinical conditions associated with muscle damage and myocardial injury.

## Introduction

Muscle damage is one of the most important factors that affects muscle fatigue during endurance exercise (Finsterer and Drory, 2016). Long-distance exercise leads to myocardial and muscle damage resulting from acute inflammatory responses and the efflux of myocellular proteins into the blood circulation increasing the risk of acute renal failure, and/or a clinically potentially life-threatening condition, rhabdomyolysis (Heled et al., 2007; Baumert et al., 2016; Finsterer and Drory, 2016; Del Coso et al., 2017). The mechanical and metabolic stress induced by exercise stimulates inflammatory cells to repair and regenerate the muscle (Hyldahl and Hubal, 2014; Peake et al., 2017).

The mechanisms involved in inflammation and muscle damage induced by exercise have been widely investigated (Peake et al., 2017). The interaction of the renin–angiotensin system (RAS) and tissue kallikrein–kinin system (TKKS) may be important to inflammatory responses and muscle homeostasis (Su, 2014; Hofman et al., 2016). Indeed, few studies have investigated the role of RAS and TKKS on inflammatory processes and muscle damage induced by endurance exercise.

Dietze and Henriksen (2008) proposed a coordinated regulation by the local RAS and TKKS, of vascular responses and glucose metabolism in oxidative muscle fibers during exercise. Bradykinin is produced by kallikrein–kinin system, an inflammatory response mechanism, binds to the bradykinin type 2 receptor (BDKRB2) and modulates signaling pathways of oxidative stress, pro-inflammatory eicosanoids and cytokines resulting in increased vascular permeability, vasodilation, hypotension, pain, and fever. Bradykinin is degraded mainly by angiotensin I-converting enzyme establishing the inter-relationship between the RAS and the TKKS which impacts muscle bioenergetic homeostasis (Hofman et al., 2016). The angiotensin-converting enzyme (ACE) catalyzes the conversion of angiotensin (ANG) I into vasoconstrictor ANG II, which can stimulate vascular smooth muscle growth, capillary density and oxygen consumption, affect both sympathetic and neuromuscular transmission and has a hypertrophic effect on skeletal muscle, improving contractile function (Cassis et al., 2002; Jones and Woods, 2003; Dietze and Henriksen, 2008; Alves et al., 2013; Vaughan et al., 2013; Vaughan et al., 2016). Moreover, ACE inﬂuences skeletal muscle function and biomechanical properties (Wagner et al., 2006).

Previous research suggested the existence of “high-responders” (HR) in accordance with high levels of muscle damage marker (creatine kinase, CK >500 UI) after resistance exercise compared to “normal responders” (NR, CK <500UI). Polymorphisms in specific genes have been linked to HR (Koch et al., 2014). It is widely accepted that physiological response phenotypes are highly polygenic (Baumert et al., 2016; Del Coso et al., 2017). The genotypes of angiotensin I-converting enzyme (*ACE*), bradykinin B2 receptor (*BDKRB2*) and angiotensinogen (*AGT*) genes have been associated with skeletal muscle efficiency, vascular response and/or muscle damage (Tobina et al., 2010; De Mello Costa and Slocombe, 2012; Popadic Gacesa et al., 2012; Eynon et al., 2014; Kikuchi and Nakazato, 2015). Recently evidence suggest that renin–angiotensin systems impact on skeletal muscle wasting (Powers et al., 2018).

Therefore, the aim of this study was to determine the extent of association between the *AGT* Met235Thr, *ACE* I/D and *BDKRB2* +9/−9 polymorphisms with inflammation, myocardial and muscle injury, induced by endurance exercise.

## Material and Methods

### Subjects

Eighty-One Brazilian male endurance runners participated in this study. Volunteers were recruited by e-mail provided by São Paulo International Marathon Organization (2015). After screening history and medical examination, 81 runners were recruited to São Paulo International Marathon 2015 (17^th^ May). We followed the same experimental procedures and design including period of blood collection and cardiopulmonary protocol as described by Santos et al. (2016).

The exclusion criteria included the use of medication to cardiac, metabolic, pulmonary, or kidney injury, use of alcohol or any kind of drugs and pathologies including systemic arterial hypertension, liver, kidney, metabolic, inflammatory, or neoplastic diseases. Subjects were informed of the experimental procedures and possible risks and signed a term of informed consent before participating, which was approved by the Ethics Committee of Dante Pazzanese Institute of Cardiology, Brazil (Permit Number: 979/2010), in accordance with the Declaration of Helsinki Measurements of total body mass (kg), height (cm) and Body Mass Index (BMI, kg/m^2^) were conducted according to the International Society for the Advancement of Kinanthropometry and expressed as the mean ± SEM.

The general and training characteristics of all marathon runners are summarized as follows: age, 39 ± 1 years; weight, 74 ± 1 kg; height, 174 ± 1 cm; BMI, 24.6 ± 0.3 kg/m^2^; training experience, 6 ± 0.5 years; time on 10 km race, 46 ± 0.7 min; frequency of training, 4.4 ± 0.7 times/week; and training volume, 56 ± 2.1 km/week.

### Cardiopulmonary Test

Anthropometrics parameters were obtained and cardiopulmonary exercise tests were performed 3 to 21 days before marathon race. Functional capacity was assessed by means of cardiopulmonary exercise test (Quark CPET, Cosmed, Rome, Italy) with expired gas analysis, performed on a treadmill (TEB Apex 200, TEB, São Paulo, Brazil, speed 0–24km/h, grade 0–35%). A protocol was used, with a starting speed of 8 km/h and grade of 1%; speed was then increased 1 km/h every 1 min. The objective was to achieve fatigue within 8 to 12 min. Blood pressure was measured with a sphygmomanometer in the beginning of the test. Respiratory gas analysis was performed by the Ergostik (Geratherm, Bad Kissingen, Germany) in breath-by-breath mode. Tests were considered maximum when at least three of these features were attained: limiting symptoms/intense physical fatigue, increase in VO_2_ lower than 2.1 ml kg^−1^ min^−1^ through an increase in the speed, attained maximal heart rate or respiratory exchange ratio higher than 1.1.

### Marathon Race

The Marathon runners were instructed to avoid pain/anti-inflammatory medication during the course of the study. The Marathon race began at 08:00 a.m. and fluid ingestion was allowed *ad libitum* during the race. Water was provided every 2 to 3 km on the running course, sports drinks on 18 and 36 km and potato or gel on 30 km. The weather parameters at São Paulo International Marathon in 2015 (TE) between 8 am to 2 pm were: average temperature 19.8 °C, maximum temperature 22.6 °C and minimum temperature 16.7 °C, average relative humidity of 72.8%, maximum relative humidity of 86% and minimum relative humidity of 61% (National Institute of Meteorology, Ministry of Agriculture, Livestock, and Supply).

### Blood Collection

Blood samples (30 ml) were collected in vacuum tubes containing an anticoagulant (0.004% EDTA) 1 day before, immediately after, 1 day, 3 days, and 15 days after São Paulo International Marathon. The marathon runners were overnight fast before blood collection on 1 day before race, 1 day after race, 3 days after race, and 15 days after race and fed immediately after race. Biochemical and genetic analyses were subsequently performed at Federal University of São Paulo, at Cruzeiro do Sul University and at Clinical Laboratory of Dante Pazzanese Institute of Cardiology. Blood samples (4 ml) were stored at −80°C for later genetic analysis after TRIZOL addition at Federal University of São Paulo and blood samples (4 ml) were centrifugated and serum separated for later cytokine analysis at Cruzeiro do Sul University.

### Biochemical Parameters

The biochemical parameters were evaluated with routine automated methodology in Clinical Laboratory of Dante Pazzanese Institute of Cardiology immediately after blood collection (22 ml). Alanine transaminase, aspartate transaminase, lactic dehydrogenase and creatine kinase were determined by kinetic assay; Troponin (I), proBNP, myoglobin, and creatine kinase-MB were evaluated by chemiluminescence assay.

The serum levels of IL-6, IL-1β, IL-10, IL-8, IL-12p70 and TNF-α were determined using BD***^™^*** Human Inflammatory Cytokine Citometric Bead Array Kit and BD Accuri cytometer according to manufacturer’s instructions (BD Biosciences, San Jose, CA, USA). The detection limit was 3.6 pg/ml for IL-8; 7.2 pg/ml for IL-1β; 2.6 pg/ml for IL-6; 3.3 pg/ml for IL-10 of; 3.7 pg/ml for TNF-α, and 1.9 pg/ml for IL12p70.

### Genetic Analysis of *AGT, ACE* and *BDKRB2* Polymorphisms

Genotyping for the identification of the *AGT* Met235Thr (rs.699) were performed using fluorescence-based TaqMan^®^ SNP Genotyping Assays (Applied Biosystems, Foster City, CA, USA). Allele specific probes and flanking primer sets were used along with a pre-made PCR master mix containing ampliTaq DNA polymerase Gold (Applied Biosystems, Foster City, CA, USA) in a reaction volume of 20 μl. PCR consisted of a 10 min heat activation step (95°C) followed by 50 cycles of 15 s at 95°C and 1 min at 60°C. Amplification was performed in PCR thermocycler, Real Time ABI 7500 (Applied Biosystems).

*ACE* insertion (I) or deletion (D) variants were screened by a polymerase chain reaction (PCR) using a sense primer (5′-CTG GAG ACC ACT CCC ATC CTT TCT-3′) and an antisense primer (5′-GAT GTG GCC ATC ACA TTC GTC AGA T-3′). The PCR product resulted in a 490 bp (I) and 190 bp (D) fragment analyzed on a 2% agarose gel stained with SYBR^®^ Safe DNA gel stain (Invitrogen).

The presence or absence of repeated sequence of 9 nucleotides of the *BDKRB2* polymorphisms were screened by a polymerase chain reaction (PCR) using a sense primer (5′-AGT CGC TCC CTG GTA CTG C-3′) and an antisense primer (5′-TCC AGC TCT GGC TTC TGG-3′). The PCR product resulted in a 89 bp (+9) and 80 bp (−9) fragment analyzed on a 4% agarose gel stained with SYBR^®^ Safe DNA gel stain (Invitrogen).

### Statistical Analyses

Statistical analyses were performed using Statistical Package for the Social Sciences (IBM SPSS Statistics for Mac, Version 24.0. Armonk, NY, USA). The Kolmogorov-Smirnov test showed the data have non-normal distribution. Differences between the steps (before, immediately after, 1 day after and 3 days after the race) in different homozygotes genotypes (DD vs II; MM vs TT; +9+9 vs −9−9) were tested for significance with Friedman test with repeated measures and Müller-Dunn post-test. Statistical significance was assumed at p-value <0.05. We also calculated the percentage of 81 runners classified as lower responders (LR), normal responders (NR) and high responders (HR) in different genotypes. The LR was classified as creatine kinase (CK) levels less 300 U/L, NR as CK levels between 300 U/L and 500 U/L and HR as levels above 500 U/L.

## Results

The genotype frequency of *AGT* Met235Thr, *ACE* I/D, *BDKRB2* +9/−9 were: TT 27.2% (22), MT 56.8% (46) and MM 16% (13) to *AGT* Met235Thr polymorphism; DD 25.9% (21), ID 58% (47) and II 16% (13) to *ACE* I/D polymorphism; and +9+9 19.8% (26), −9+9 48.1% (39), −9−9 32.1% (16) to *BDKRB2* +9/−9 polymorphism. All the observed genotype frequencies were consistent with Hardy-Weinberg equilibrium (*AGT* Met235Thr, p = 0.177; *ACE* I/D, p = 0.122; *BDKRB2* +9/−9, p = 0.842).

The general and training characteristics were similar between different *ACE, AGT* and *BDKRB2* genotypes (**Table 1**).

**Table 1:** General and training characteristics of marathon runners separated by genotype.

ACE I/D	DD	ID	II
**Age (years)**	39 ± 6.8	38 ± 9.3	42 ± 8.8
**Weight (kg)**	77 ± 10.5	74.2 ± 9.7	70.7 ± 8.8
**Height (m)**	1.74 ± 0.08	1.74 ± 0.06	1.74 ± 0.06
**BMI (kg/m^2^)**	25.4 ± 2.52	24.6 ± 2.54	23.3 ± 2.45
**TE (years)**	6.3 ± 5.7	6.1 ± 4.5	5.7 ± 3.5
**Training (km/week)**	59.1 ± 16.5	56.2 ± 21.4	53.5 ± 20.7
**FT (time/week)**	4.7 ± 1.2	4.4 ± 1.3	3.8 ± 0.9
**10 km race (min)**	46.5 ± 6.2	45.8 ± 6.1	45.7 ± 5.2
**VO_2_ peak (ml/kg/min)**	58.04 ± 5.24	57.04 ± 6.40	58.01 ± 8.15
**Race time (min)**	247.6 ± 30.9	264.2 ± 45.3	253.7 ± 34.9
***AGT Met235Thr***	***MM***	***MT***	***TT***
**Age (years)**	33 ± 9.7	40 ± 7.4	40 ± 9.5
**Weight (kg)**	71.8 ± 8.8	75.6 ± 10.7	73.5 ± 8.5
**Height (m)**	1.72 ± 0.04	1.74 ± 0.07	1.74 ± 0.07
**BMI (kg/m^2^)**	24.1 ± 2.34	24.9 ± 2.55	24.2 ± 2.7
**TE (years)**	5.3 ± 2.6	5.4 ± 3.6	7.8 ± 6.64
**Training (km/week)**	61.5 ± 31.8	56.5 ± 16.2	54 ± 19.3
**FT (time/week)**	4.5 ± 1.3	4.3 ± 1.4	4.3 ± 1.1
**10 km race (min)**	43.5 ± 4	47.7 ± 6.2	44 ± 5.7
**VO_2_ peak (ml/kg/min)**	58.51 ± 6.93	56.03 ± 5.61	59.6 ± 6.89
**Race time (min)**	267.1 ± 37.83	251.9 ± 38.04	268.4 ± 48.24
***BDKRB2 +9/−9***	***−9/−9***	***−9+9***	***+9+9***
**Age (years)**	36 ± 7	40 ± 6.4	38 ± 9.3
**Weight (kg)**	73.3 ± 9.2	76 ± 10.1	72.4 ± 10.2
**Height (m)**	1.72 ± 0.09	1.74 ± 0.06	1.74 ± 0.05
**BMI (kg/m^2^)**	24.4 ± 2.33	25.0 ± 2.76	24.3 ± 2.42
**TE (years)**	5.5 ± 4.4	6.1 ± 4.8	7.1 ± 4.8
**Training (km/week)**	57.2 ± 24.7	57 ± 17.5	54.1 ± 18.2
**FT (time/week)**	4.2 ± 1.3	4.4 ± 1.3	4.6 ± 1.3
**10 km race (min)**	46.2 ± 4.7	45.2 ± 5.7	47.8 ± 8.4
**VO_2_ peak (ml/kg/min)**	55.22 ± 5.93	58.90 ± 6.61	57.39 ± 5.61
**Race time (min)**	259.4 ± 40.76	252.4 ± 42.3	274.4 ± 36.49

ACE, angiotensin I-converting enzyme; AGT, angiotensinogen; BDKRB2, bradykinin B2 receptor; TE, training experience; and FT, frequency of training. The values presented are the mean ± SD of 13-47 runners.

Considering all runners, the marathon race induced an increase in myoglobin (22-fold, p < 0.0001), CK (7.2-fold, p < 0.0001), Pro-BNP (2.5-fold p < 0.0001), troponin (4-fold p < 0.0001), CK-MB (7.6-fold, p < 0.0001), LDH (2-fold, p < 0.0001), AST (2.6-fold, p < 0.0001), ALT (1.4-fold, p < 0.0001), leukocytes (2.6-fold, p < 0.0001), neutrophils (3.9-fold, p < 0.0001), monocytes (1.8-fold, p < 0.0001), IL-6 (5.3-fold, p < 0.0001), IL-8 (1.8-fold, p < 0.05), IL-1β (1.9-fold, p < 0.0001) and IL-10 (3-fold, p < 0.0001) (**Table 2**). The lymphocyte count decreased by 35% immediately after race (**Table 2**). Troponin I concentration returned to baseline one day after race; myoglobin level three days after race; LDH, CK, AST and pro-BNP levels 15 days after race; and CK-MB and ALT remained elevated 15 days after race (**Table 2**). All cytokines and lymphocyte count numbers returned to baseline one day after race (**Table 2**). Neutrophils and monocytes returned to baseline levels 3 days after race (**Table 2**).

**Table 2:** Markers of inflammation and muscle damage after marathon race.

	Before	After	1 day after	3 days after	15 days after
**Myoglobin (ng/ml)**	46 ± 50	1047 ± 702^**^	184 ± 157^**^	69 ± 104	71 ± 149
**LDH (U/L)**	480 ± 104	955 ± 267^**^	681 ± 174^**^	606 ± 147^**^	517 ± 139
**CK (U/L)**	286 ± 735	679 ± 1151^**^	2070 ± 2012^**^	915 ± 1670^*^	461 ± 1985
**CK-MB (µg/L)**	1.7 ± 1.8	4.9 ± 3.7^**^	13.2 ± 13.7^**^	3.3 ± 2.4^*^	2.3 ± 1.5*
**AST (U/L)**	36 ± 12	51 ± 20^**^	93 ± 53^**^	65 ± 36^**^	39 ± 36
**ALT (U/L)**	39 ± 12	43 ± 14^*^	48 ± 14^**^	52 ± 17^**^	48 ± 23*
**Troponin (ng/ml)**	0.02 ± 0.01	0.09 ± 0.02^*^	0.03 ± 0.05	0.02 ± 0.02	0.02 ± 0.01
**Pro BNP (pg/ml)**	38 ± 24	115 ± 90^*^	90 ± 54**	48 ± 36*	41 ± 24
**IL-6 (pg/ml)**	3.1 ± 2.1	16.3 ± 16.2**	3.7 ± 2	4.1 ± 1.8	ND
**IL-8 (pg/ml)**	4.4 ± 2	7.9 ± 7.5*	4.5 ± 1.5	5.5 ± 8.1	ND
**IL-1β (pg/ml)**	4.6 ± 0.3	8.6 ± 6.3**	5.3 ± 2.4	5.2 ± 2	ND
**IL-10 (pg/ml)**	3 ± 1	8.6 ± 7.1**	3.2 ± 1.1	3.6 ± 2.2	ND
**TNF-α (pg/ml)**	4.9 ± 4.1	4.9 ± 4.3	5.1 ± 5.3	5.2 ± 5	ND
**IL-12p70 (pg/ml)**	3 ± 2.3	5 ± 6.2	3 ± 2.3	3.3 ± 2.6	ND
**Leukocytes (×10^3^mm^3^)**	6 ± 1.3	15.5 ± 3.4**	7.2 ± 1.6**	5.5 ± 1	6 ± 1.4
**Neutrophils**	3.4 ± 1	13.4 ± 3.3**	4.6 ± 1.3**	3.1 ± 0.8	3.6 ± 1
**Monocytes**	0.4 ± 0.1	0.7 ± 0.4**	0.5 ± 0.2**	0.35 ± 0.1	0.35 ± 0.1
**Lymphocytes**	2 ± 0.5	1.3 ± 0.5**	1.9 ± 0.5	1.8 ± 0.4	1.9 ± 0.5

LDH, lactate dehydrogenase; CK, creatine kinase; ALT, alanine transaminase; AST, aspartate transaminase; NT-BNP, N terminal pro-B-type natriuretic peptide; ND, not determined; IL, interleukin; TNF, Tumor necrosis factor. Values are means ± SD of 81 runners. *P < 0.05 vs. before the race; **p < 0.0001 vs. before the race.

### *ACE* I/D

**Figure 1:**
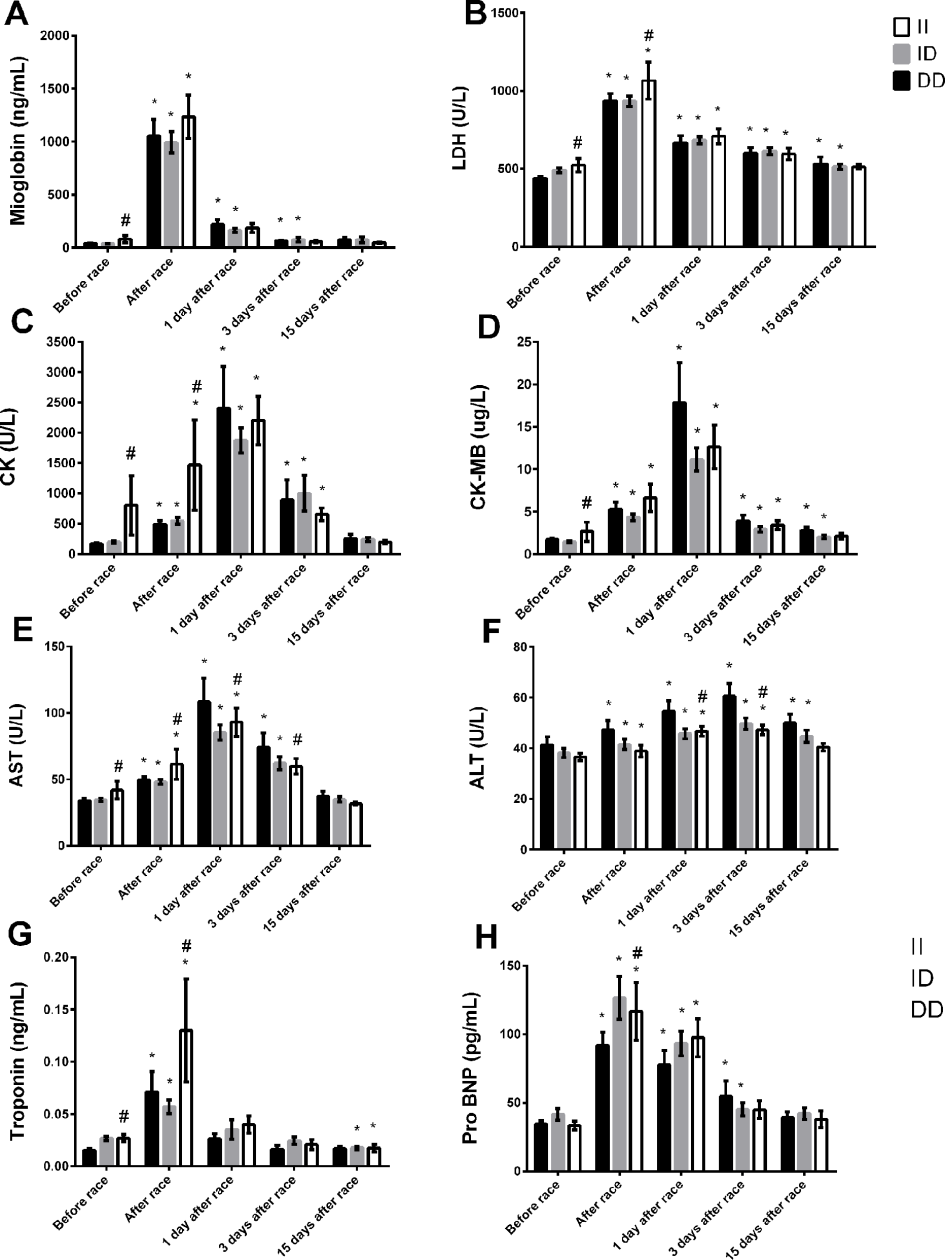
Effect of marathon on markers of muscle damage in DD, ID and II ACE genotypes. The markers of muscle damage evaluated were myoglobin **(A)**; lactate dehydrogenase, LDH, **(B)**; creatine kinase, CK **(C)**; CK-MB **(D)**; aspartate transaminase, AST **(E)**; alanine transaminase, ALT **(F)**; troponin **(G)**; brain natriuretic peptide, Pro BNP **(H)**. The values are presented as of mean ± SEM of 13–47 runners. *P < 0.05 vs before; ^#^P < 0.05 II vs DD genotype.

The baseline levels (before race) of myoglobin and troponin, LDH, CK, CK-MB, and AST were slightly higher in II genotype compared to DD genotypes (**Figures 1A–D, F, G**). Immediately after the race, the myocardia damage markers, troponin and pro BNP and muscle damage markers, CK and LDH levels were also higher in II genotype (**Figures 1A, C, H**). However, AST and ALT activities were lower in II genotype 1 and 3 days after the race (**Figures 1E, F**). Moreover, LDH, CK-MB and ALT returned to basal levels 15 days after race in II genotype but not in DD genotype, probably due higher levels of these markers on baseline in II genotypes (**Figures 1A, D, F**). We also observed a reduction in troponin levels in II genotypes 15 days after race compared to baseline levels (**Figure 1G**).

The percentage of runners high responders (>500U/I) to CK levels was 38% for DD genotypes, 40% to ID genotypes and 69% for II genotypes immediately after race.

**Figure 2:**
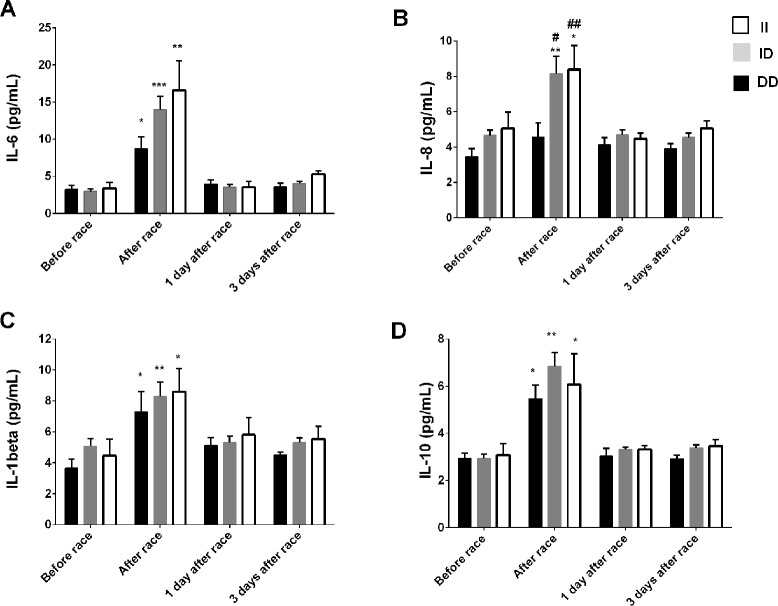
Effect of marathon on cytokines in DD, ID and II ACE genotypes. The inflammatory mediators evaluated were interleukin (IL)-6 **(A)**; IL-8 **(B)**; IL-1β **(C)**; IL-10 **(D)**. The values are presented as of mean ± SEM of 9–33 runners. ^*^P < 0.05 vs before;** P < 0.0001 vs before; ^#^P < 0.05 vs II genotype; ^##^P < 0.0001 vs II genotype.

Immediately after the race, we reported an increase in IL-8 levels just in ID and II genotypes and a pronounced response on IL-6, IL-10 and IL-1β levels in ID and II genotypes, however, changes were not significant (**Figure 2**). We did not find differences in leukocyte count between *ACE* I/D genotypes (data not shown), on TNF-α and IL-12p70 concentrations after the marathon race and between ACE I/D genotypes (data not shown).

### *AGT* Met235Thr

**Figure 3:**
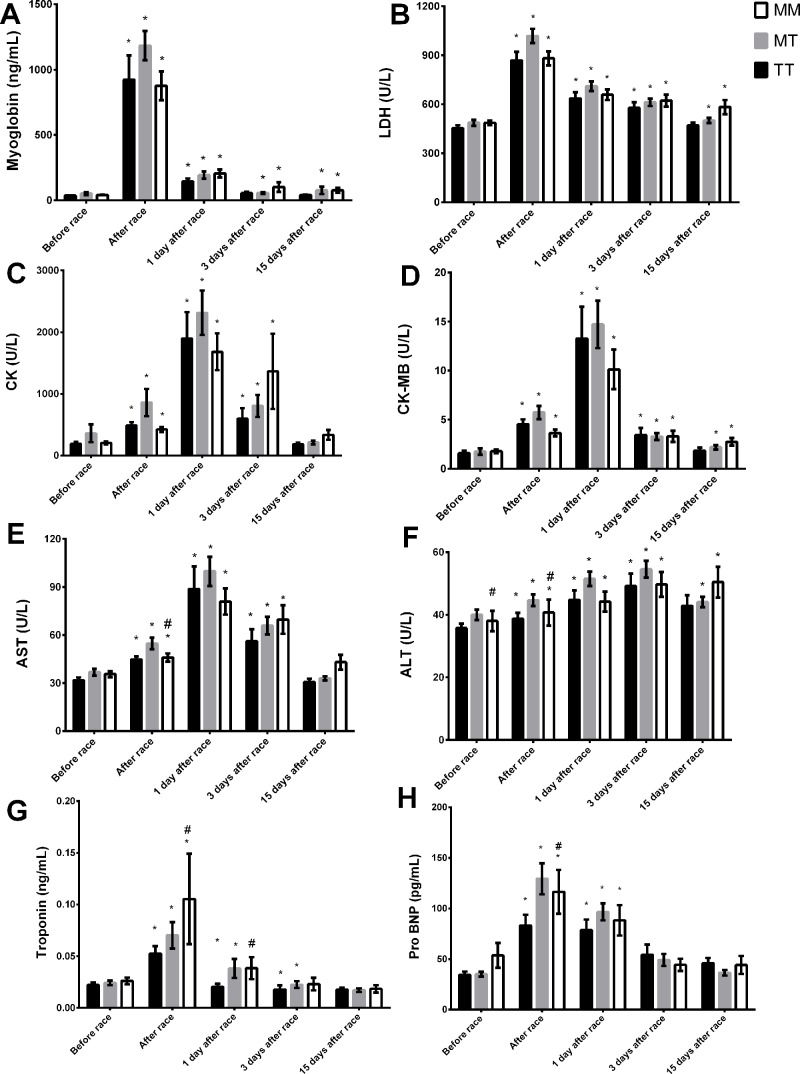
Effect of marathon on markers of muscle damage in MM, MT and TT AGT genotypes. The markers of muscle damage evaluated were myoglobin **(A)**; lactate dehydrogenase, LDH, **(B)**; creatine kinase, CK **(C)**; CK-MB **(D)**; aspartate transaminase, AST **(E)**; alanine transaminase, ALT **(F)**; troponin **(G)**; brain natriuretic peptide, Pro BNP **(H)**. The values are presented as of mean ± SEM of 16–43 runners. *P < 0.05 vs before; ^#^P < 0.05 MM vs TT genotype.

Troponin and pro BNP response were more pronounced in MM genotype compared to TT genotype immediately and/or one day after race (**Figures 3G, H**). However, we observed lower ALT activity before and immediately after race in MM genotype compared to TT genotype (**Figure 3F**). Myoglobin level and LDH and CK-MB activities recovered in MM homozygotes but remained altered in TT genotype 15 days after race (**Figures 3A, B, D**). CK, troponin, ALT and AST levels returned to baseline levels 15 days after race in all genotypes (**Figures 3C, E–G**).

**Figure 4:**
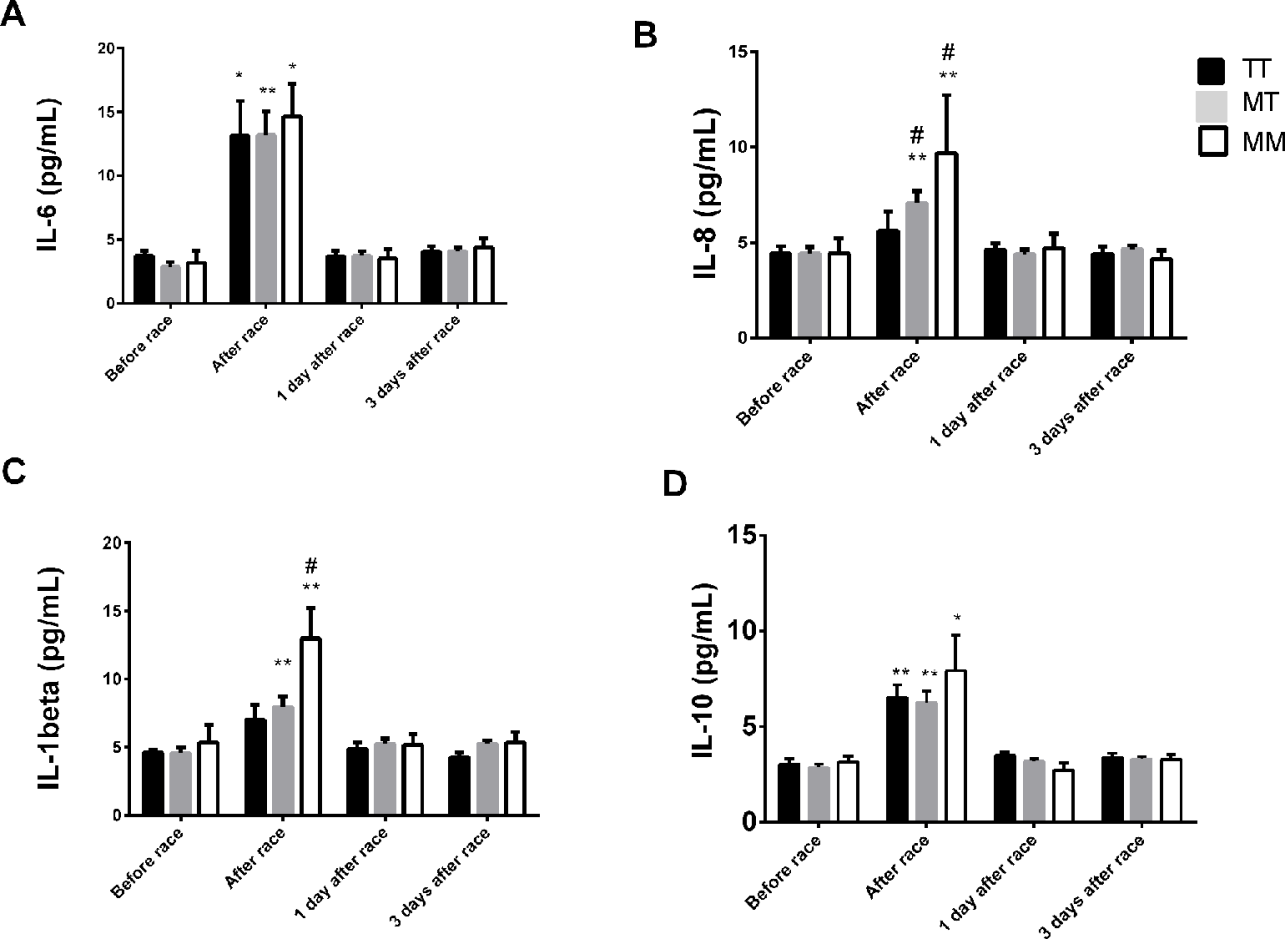
Effect of marathon on cytokines in MM, MT and TT AGT genotypes. The inflammatory mediators evaluated were interleukin (IL)-6 **(A)**; IL-8 **(B)**; IL-1β **(C)**; IL 10 **(D)**. The values are presented as of mean ± SEM of 9–33 runners. *P < 0.05 vs before; ** P < 0.0001 vs before; ^#^P < 0.05 MM vs TT genotype.

IL-6 and IL-10 concentration increased after race in all *AGT* Met235Thr genotypes (**Figures 4A, D**). The Marathon race induced an elevation on IL-1β and IL-8 levels on MT and MM genotypes but not in TT genotypes (**Figures 4B, C**). No differences were observed on TNF-α and IL-12p70 levels after marathon race and between *AGT* Met235Thr genotypes (data not shown).

### *BDKRB2* +9/−9

**Figure 5:**
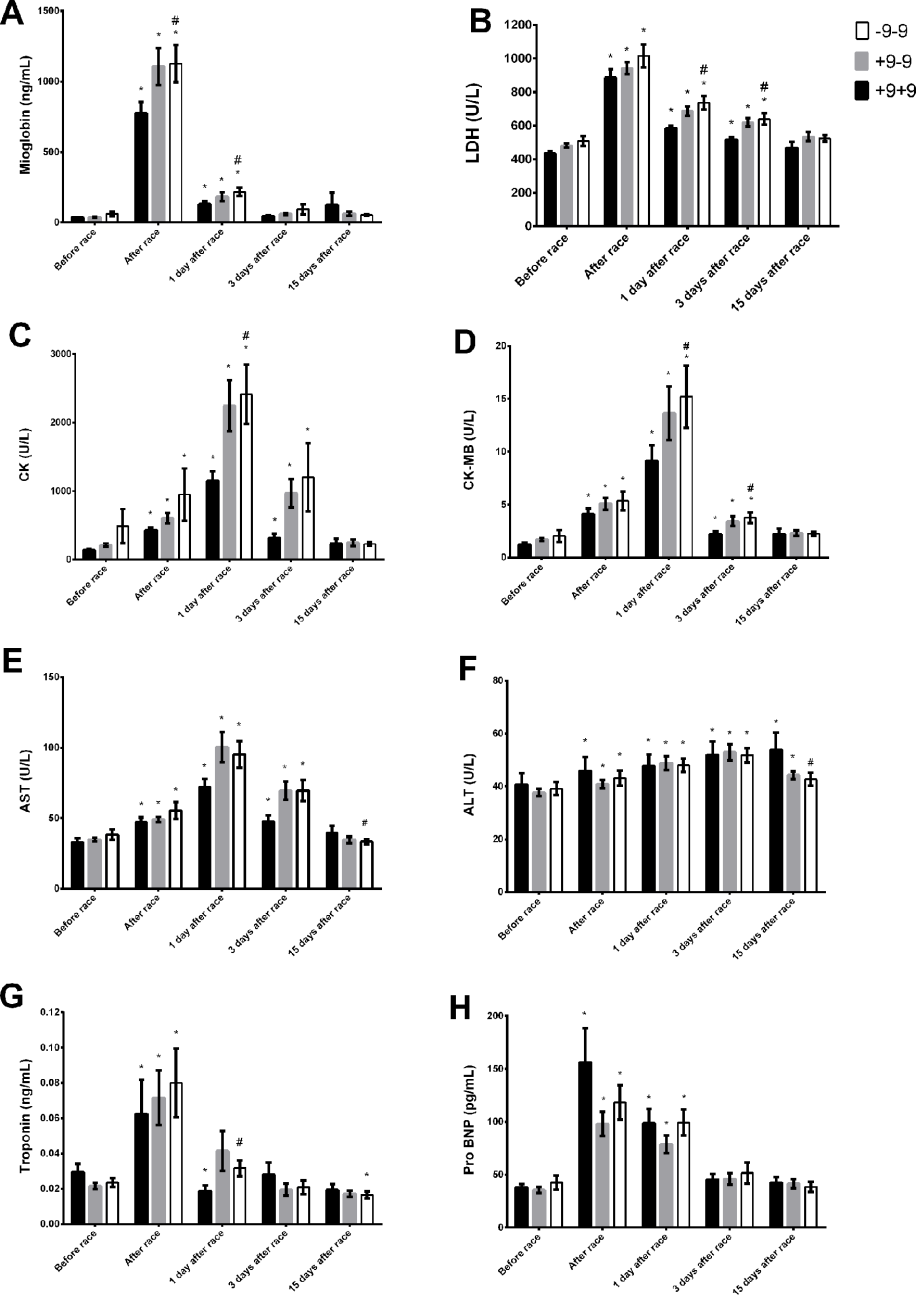
Effect of marathon on markers of muscle damage in +9+9, −9+9 and −9/−9 BDKRB2 genotypes. The markers of muscle damage evaluated were myoglobin **(A)**; lactate dehydrogenase, LDH, **(B)**; creatine kinase, CK **(C)**; CK-MB **(D)**; aspartate transaminase, AST **(E)**; alanine transaminase, ALT **(F)**; troponin **(G)**; brain natriuretic peptide, Pro BNP **(H)**. The values are presented as of mean ± SEM of 16–39 runners. *P < 0.05 vs before; ^#^P < 0.05 −9/−9 vs +9+9 genotype.

*BDKRB2* −9−9 were associated with elevated LDH, CK, CK-MB, and ALT and AST activities and myoglobin and troponin levels immediately, 1 day and/or 3 days after race (**Figures 5A–G**). ALT levels increased 15 days after race in +9+9 genotypes and returned to basal levels in −9−9 genotypes (**Figure 5F**). ALT and AST activities were lower in −9−9 genotypes 15 days after race compared to +9+9 genotypes (**Figures 5E, F**). Pro-BNP increased immediately after race and returned to baseline 3 days after race in all BDKRB2 genotypes (**Figure 5H**).

The percentage of runners higher responders (>500U/I) to CK levels was 31% for +9+9 genotypes, 48% for −9+9 genotypes and 46% for −9/−9 genotypes immediately after race.

**Figure 6:**
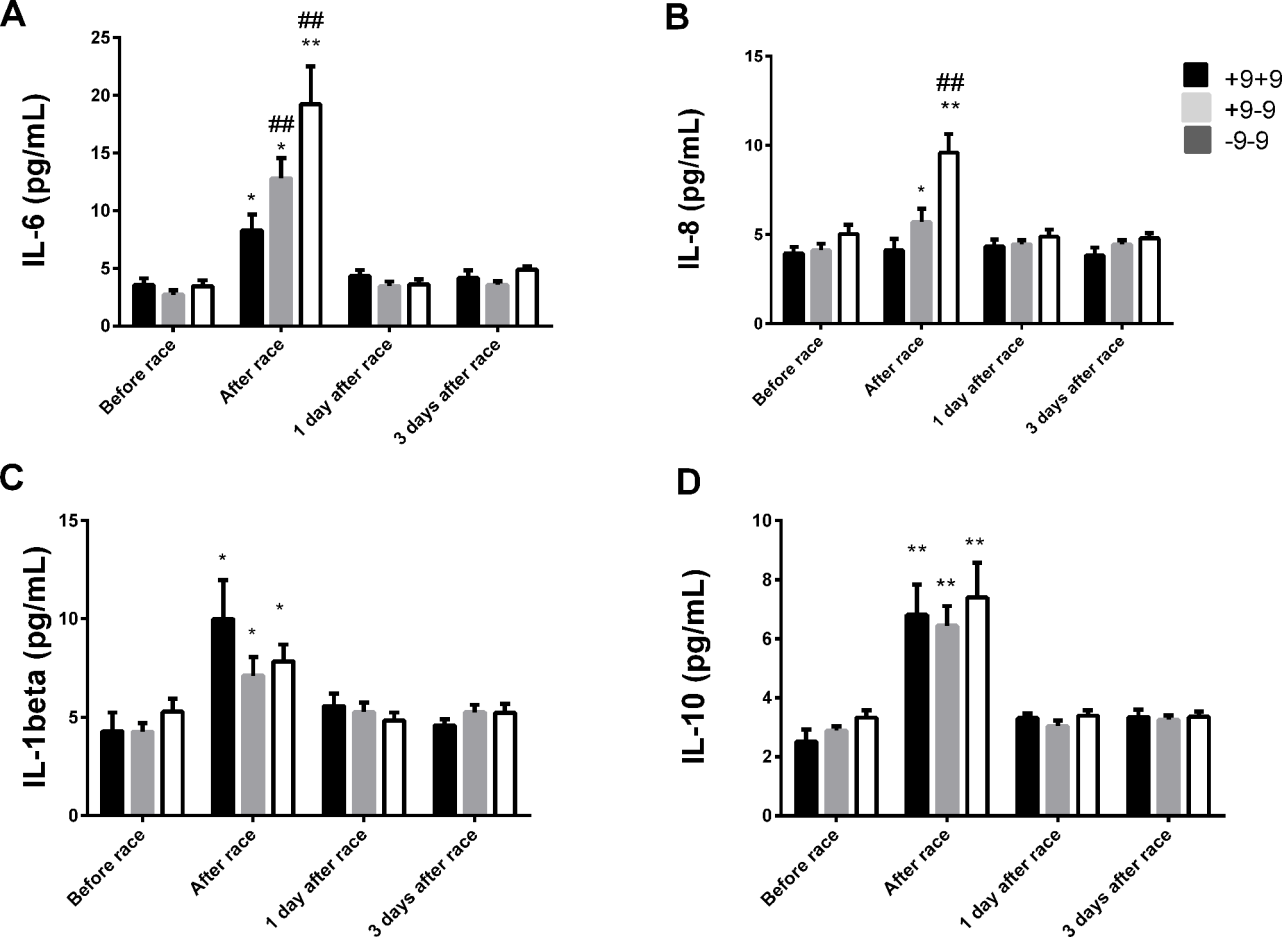
Effect of marathon on cytokines in +9+9, −9+9 and −9/−9 BDKRB2 genotypes. The inflammatory mediators evaluated were interleukin (IL)-6 **(A)**; IL-8 **(B)**; IL-1β **(C)**; IL-10 **(D)**. The values are presented as of mean ± SEM of 9–33 runners. *P < 0.05 vs before; ** P < 0.0001 vs before; ^##^P < 0.0001 vs +9+9 genotype.

IL-6 levels were elevated in all genotypes, but were more pronounced in −9−9 genotypes (**Figure 6A**). Immediately after race, we observed an increase on IL-8 levels in −9+9 and −9−9 genotypes and a pronounced response in −9−9 genotypes compared to −9+9 genotypes (**Figure 6B**). The Marathon race also promoted IL-10 and IL-1β release to the same extent between *BDKRB2* +9/−9 genotypes (**Figures 6C, D**). We did not find differences in TNF-α and IL-12p70 concentrations after the marathon race as also found between *BDKRB2* +9/−9 genotypes (**Figure 6**).

## Discussion

The results described herein suggest that *ACE* II and *BDKRB2* −9−9 genotypes are associated with higher skeletal muscle and myocardial injury and inflammation and *AGT* MM genotype is associated with greater myocardial injury and inflammation. The *ACE* DD, *AGT* TT and *BDKRB2* +9+9 genotypes are additionally associated with low inflammatory response and muscle damage confirming that RAS and KKS play an important role in inflammation and muscle damage induced by exercise.

There was no difference of the genotype frequencies according with Hardy-Weinberg equilibrium. The Brazilian population is one of the most mixed in the world, which is an outcome of post-Columbian admixture between Amerindians, Europeans colonizers or immigrants, and African slaves (Kehdy et al., 2015). The equilibrium of genotype sample can gives an idea of non-based sample randomization.

The *ACE* D allele has been associated with greater muscle power phenotypes after resistance exercise (Erskine et al., 2014; Freire et al., 2015). In our study the muscle damage markers measured before the race and CK and LDH levels measured immediately after the race were also lower in DD homozygotes, similar to results previously reported (Amir et al., 2007). The higher muscle damage susceptibility may be related to the inflammatory process modulated by RAS and KKS. The lower ACE activity promotes higher levels of bradykinin, which induces local inflammation, vasodilation, and extravasation of myocellular proteins (Hofman et al., 2016). Our results describe a pronounced inflammatory response due to muscle damage in II genotype individuals (low ACE activity) and in the athletes carrying the −9/−9 genotype (high bradykinin activity) demonstrating the association of RAS and KKS with the inflammatory process and muscle damage induced by exercise. There are multiple cross-talk points between the RAS and the KKS that can occur in the same or opposite direction. The tissue damage may upregulate both the RAS and KKS (Su, 2014).

The kallikrein–kinin system involves the cleavage of high molecular weight kininogen with release of bradykinin and antimicrobial peptides (AMPs), linked to the intrinsic coagulation system *via* factor XI (FXI) (Hofman et al., 2016). The endogenous activators of this contact system are polyphosphate, collagen, misfolded protein, aggregates, lipopolysaccharides (LPS), glycosaminoglycans, and nucleic acids and phosphatidylserine (Wu, 2015). Previous studies of our group demonstrated lymphocyte phosphatidylserine externalization after endurance exercise (Santos et al., 2013) which could activates KKS. Moreover, Meotti et al. (2012) demonstrated that inﬂammatory muscle involves the synthesis of IL-1β, TNF-α and IL-6 associated with the up-regulation of both B1 and B2 kinin receptors (Meotti et al., 2012). Chen et al. (2017) proposed that −9−9 genotypes have a pronounced pro-inflammatory response in inflammatory animal models (Chen et al., 2017). The blockage of BDKRB2 also improves inflammatory responses in skin wound healing in diabetic mice (Desposito et al., 2016). The higher production of NO in −9−9 genotypes also could inhibit mitochondrial complex IV and reduce oxidative metabolism and availability of ATP (Chen et al., 2017). Thus, we suggest that the attenuation on muscle damage in individuals with the +9+9 genotype, which has lower transcriptional activity in relation to BDKRB2, may be related to lower inflammatory responses after the marathon race.

Muscle damage and inflammatory responses induced by exercise could be influenced by metabolic stress. Individuals carrying the II genotype have higher percentage of type I skeletal fiber while individuals with the DD genotype have a higher percentage of IIb skeletal fiber (Tobina et al., 2010), supported by previous studies reporting a local improvement in metabolism and capillarization during recovery training in II homozygotes (Dietze and Henriksen, 2008; Vaughan et al., 2013; Vaughan et al., 2016). Kinin B2 receptor expression is associated with increased skeletal muscle glucose uptake, greater blood flow in muscles and higher metabolic skeletal muscle contraction and efficiency (Williams et al., 2004). Blood flow and vascular conductance promoted by exercise training appear to be greater in *BDKRB2* −9/−9 homozygotes (Alves et al., 2013). However, the higher metabolic efficiency of −9/−9 and II genotypes appear not to be associated with muscle damage. Moreover, the II genotype demonstrated lower levels of AST and ALT in the recovery period, which may be attributed to the variability in the proportion of skeletal muscle type I and IIa or IIb and the pronounced metabolic efficiency of type I skeletal muscle.

RAS and KKS systems also play an important role in cardiovascular systems (Su, 2014). We also have reported that DD, +9+9 and TT genotypes are associated with lower myocardial damage markers levels, as assessed by troponin and/or pro BNP markers. Expression of the D allele was associated with higher ejection fractions; right ventricular diameter in diastole and pulmonary artery systolic pressure in football players (Saber-Ayad et al., 2014). Expression of *AGT* T allele is also associated with left ventricular hypertrophy and enhanced production of ANG II, which mediates vascular smooth muscle growth, capillary density and oxygen consumption in muscle (Cassis et al., 2002; Jones and Woods, 2003). In addition, ANG II is an important contributor to cardiac and vascular remodeling (Unger, 2002) and may contribute to lower levels of myocardial injury (troponin and Pro BNP levels) after the race in TT genotypes. +9+9 genotypes also contributed to left ventricular hypertrophy (Su, 2014). These data indicate that RAS and KKS also are related to myocardial damage.

Our results indicate slower recovery of myocardial damage and lower levels of inflammatory mediators after endurance exercise in DD genotypes (pro BNP). It may suggest that myokines (IL-6 and IL-8) play an important role in the recovery of myocardial damage.

These data suggested that *ACE* II, *AGT* MM and *BDKRB2* −9/−9 genotypes were associated with higher skeletal muscle and/or myocardial injury and inflammation. The pronounced inflammatory response is associated with RAS and KKS which could contribute to elevated risk with respect to muscle and myocardial damage and/or to repair and recovery of the tissue damage in *ACE* II, *AGT* MM and *BDKRB2* −9/−9 genotypes.

Thus, in summary, our findings demonstrated that genetic polymorphisms related to RAS and KKS system may be included in the evaluation of inflammatory and muscle damage risks in amateur athletes. This may be a novel and additional tool for coaches, in order to improve the specificity of the training program, considering aspects like intensity, recovery and/or supplementation. It could be beneficial to avoid an exacerbated muscle and myocardial damage in susceptible individuals during endurance training/competitions.

## Ethics Statement

This study was carried out in accordance with the recommendations of 'name of guidelines, name of committee' with written informed consent from all subjects. All subjects gave written informed consent in accordance with the Declaration of Helsinki. The protocol was approved by the “Ethics Committee of Dante Pazzanese Institute of Cardiology, Brazil (Permit Number: 979/2010).

## Author Contributions

AS carried out data collection, participated in its design, performed the statistical analysis, and helped draft the manuscript; MB, JM, RO and PM were responsible for data collection. GL and ES carried out the genetic analysis; MK, JP, NG, and PN participated in experimental design and coordination and helped to draft the manuscript; MC-B conceived of the study, participated in its design and coordination, helped to perform the statistical analysis and drafted the manuscript. All authors have read and approved the final version of the manuscript, and agree with the order of presentation of the authors.

## Funding

This work was supported by Fundação de Amparo à Pesquisa do Estado de São Paulo (FAPESP) [Grant Number 2014/21501-0].

## Conflict of Interest

The authors declare that the research was conducted in the absence of any commercial or financial relationships that could be construed as a potential conflict of interest.
